# Transverse tibial bone transport to avoid major lower limb amputation: A case report

**DOI:** 10.1016/j.ijscr.2025.111508

**Published:** 2025-06-13

**Authors:** Adrienne Estes, Mikhail Samchukov

**Affiliations:** a Western University of Health Sciences, College of Podiatric Medicine, Pomona, CA, USA; b Center for Excellence in Limb Lengthening and Reconstruction, Texas Scottish Rite Hospital for Children, Dallas, TX, USA

**Keywords:** Transverse tibial bone transport, Ischemic foot, Limb salvage, Ilizarov, Distraction osteogenesis

## Abstract

**Introduction & importance:**

The use of transverse tibial bone transport has gained recognition as a novel intervention for limb salvage and an effective method for managing diabetic ischemic wounds.

**Case presentation:**

This is a case report of an ischemic foot subject to potential below the knee amputation. Following attempted failed vascular intervention, the patient underwent a transverse tibial bone transport that was successful in avoiding a below the knee amputation.

**Clinical discussion:**

Transverse tibial bone transport is an innovative approach to limb salvage surgery that has shown significant promise in improving outcomes of dysvascularized feet, particularly in patients with critical limb ischemia (CLI) and those at risk of amputation.

**Conclusion:**

By leveraging the principles of distraction osteogenesis, the facilitated neovascularization through transverse tibial bone transport can have a crucial impact on healing in dysvascular conditions.

Level of evidence: IV.

## Highlights

• Innovative Approach to Ischemic diabetic feet
• Enhanced bone regeneration and healing
• Reduced risk for major limb amputations
• Neovascularization through external fixation distraction osteogenesis

## 1 Introduction

Diabetic ischemic ulcers are a significant complication of diabetes mellitus, arising primarily from a combination of peripheral neuropathy, poor circulation, and chronic hyperglycemia [1]. These ulcers typically develop on the feet and lower extremities and are often exacerbated by the presence of peripheral arterial disease (PAD), which limits blood flow and impairs wound healing [2,3]. The pathophysiology of diabetic ulcers involves a complex interplay of factors, including impaired immune response, neuropathic changes, and microvascular complications, which collectively contribute to the high morbidity associated with these wounds [4].

The prevalence of diabetic foot ulcers is alarmingly high, affecting approximately 15 % of individuals with diabetes during their lifetime, with a substantial proportion leading to infections and even lower extremity amputation [5]. This situation is particularly concerning given the increasing incidence of diabetes globally, making diabetic ulcers a major public health challenge [6].

Traditional management for diabetic ischemic ulcers includes optimizing glycemic control, providing appropriate wound care, and addressing ischemia through revascularization techniques when feasible [7]. However, in cases where ischemic ulcers result in nonhealing wounds and traditional treatments are insufficient, advanced surgical options, such as transverse tibial bone transport, may be considered. The procedure involves the use of distraction osteogenesis to regenerate bone and soft tissue, facilitating the reconstruction of defects and improving the vascularization of the affected area [[8], [9], [10], [11], [12]].

## 2 Case presentation

**Fig. 1:**
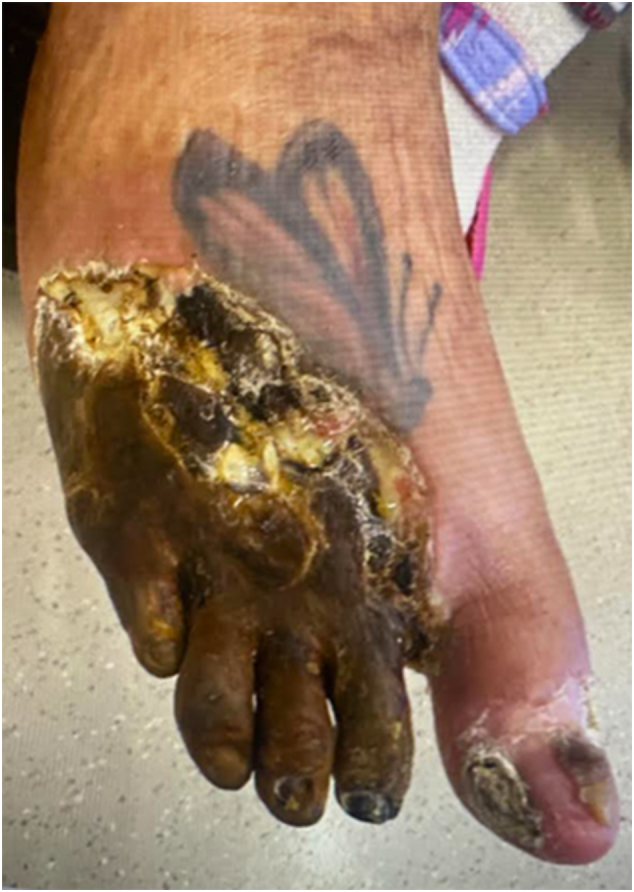
Preoperative clinical picture during initial hospital encounter.

**Fig. 2:**
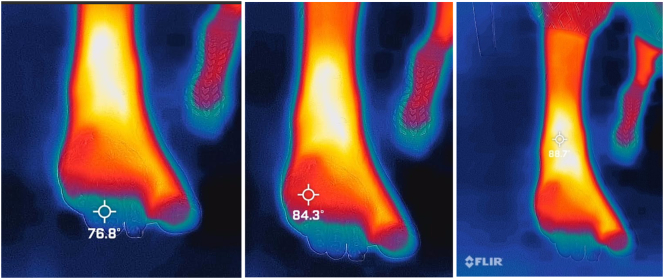
FLIR thermal images demonstrating absence of blood flow to lesser digits.

**Fig. 3:**
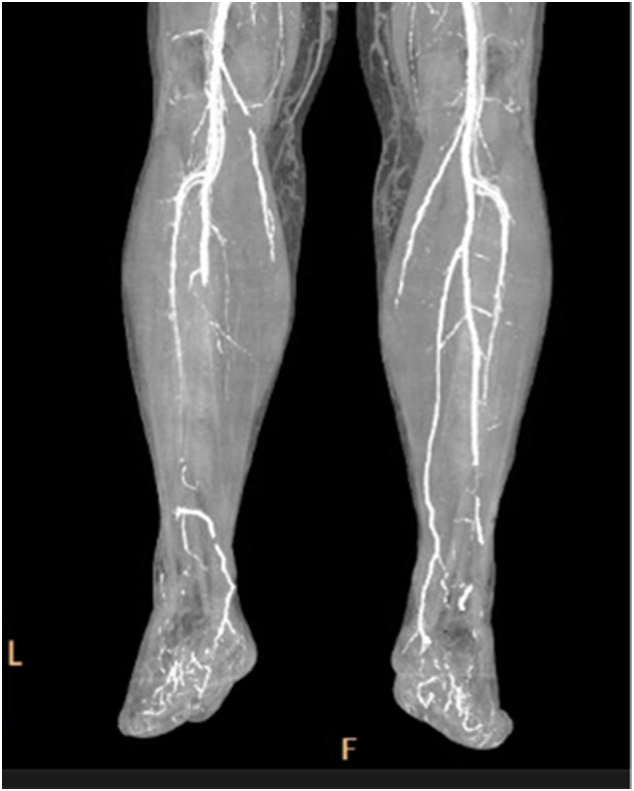
CT angiogram demonstrating ATA/PTA occlusion and Peroneal Artery stenosis.

A 54-year-old female with a past medical history significant for IDDM2, hypothyroidism, and hypertension presented with a right lateral gangrenous foot and poor blood flow, as evidenced by clinical examination, thermal images and CT angiogram (Fig. 1, Fig. 2, Fig. 3).

A preoperative peripheral angiogram demonstrated occlusion of the distal ATA/PTA and peroneal artery stenosis. Transcutaneous oxygen pressure testing revealed reads of 3 mmHg to the forefoot, 13 mmHg to the hindfoot and 37 mmHg to below the knee.

Revascularization was attempted, but there was an inadequate response in blood flow below the ankle, with a poor prognosis. One to three weeks after this attempt, a repeat transcutaneous oxygen test was performed, showing continued poor healing potential. Given only one vessel run off, the patient was not a candidate for a free flap or other vascular-targeted reconstruction options per vascular and plastic surgery consultations. The patient opted to avoid a below-the-knee amputation and agreed to undergo a transverse tibial bone transport for lower limb salvage alongside a distal amputation of the gangrenous forefoot.

## 3 Surgical technique

**Fig. 4:**
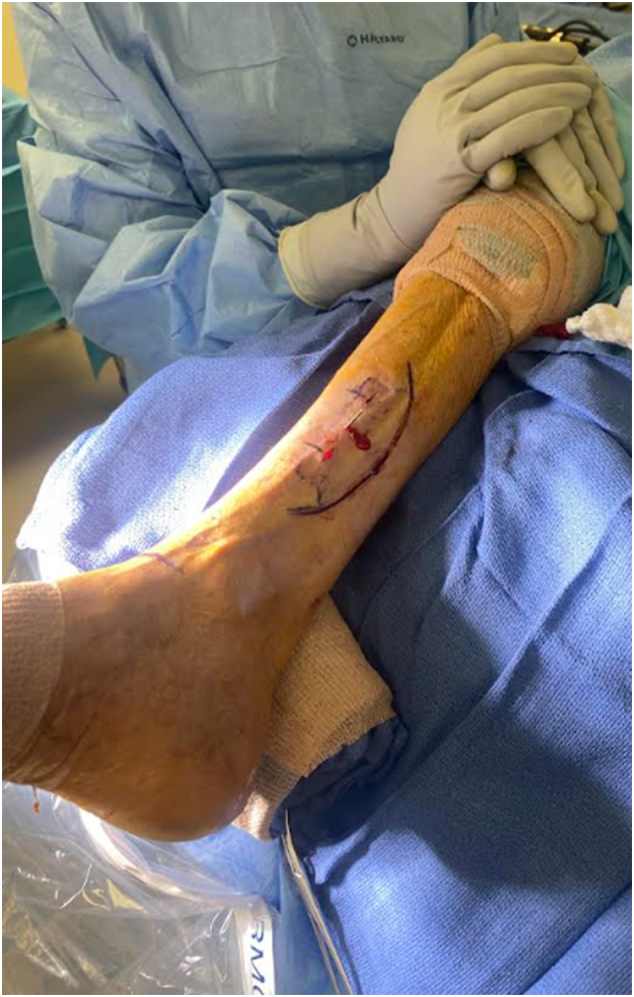
Incision outline for transverse tibial bone transport.

The transverse tibial bone transport was performed through an incision approximately 10 cm long, arched posteriorly along the medial face of the distal tibia. Using a template outline and fluoroscopy to guide the planned osteotomy, we ensured the template was positioned at least 20 mm from the ankle joint to accommodate the placement of stabilizing bicortical half pins (Fig. 4).

**Fig. 5:**
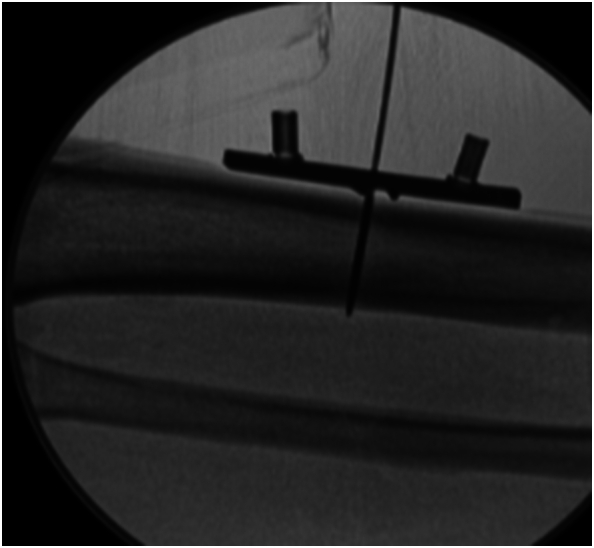
Template position secured with 1.8 mm central bicortical stabilization wire.

The template was secured directly through the skin using a 1.8 mm diameter bicortical wire through the central hole of the template (Fig. 5). The skin was then marked around the perimeter of the template, as well as at the incision sites for the anticipated guide tubes and unicortical transport segment half pins. The template was then removed, leaving the central wire intact, and initial dissection to the bone was performed with care to preserve the periosteum.

**Fig. 6:**
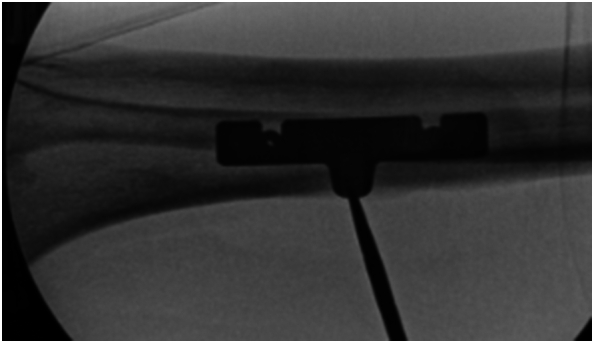
Template repositioning on the central bicortical wire.

Next, we repositioned the template over the existing central stabilization wire and confirmed the template's orientation using fluoroscopy (Fig. 6).

At this point, the template was further stabilized with two additional bicortical wires before performing the corticotomy. The boundaries of the template were perforated with 2.7 mm pilot drill holes to create a rectangular corticotomy. The corticotomy was then partially completed using a sagittal saw along the proximal, distal, and anterior aspects of the predrilled rectangular area. After skin repositioning and using a 2.7 mm drill bit, the planned half-pin sites were predrilled through the guide tubes on the template followed by insertion of two converging 4 mm diameter transport segment half pins.

**Fig. 7:**
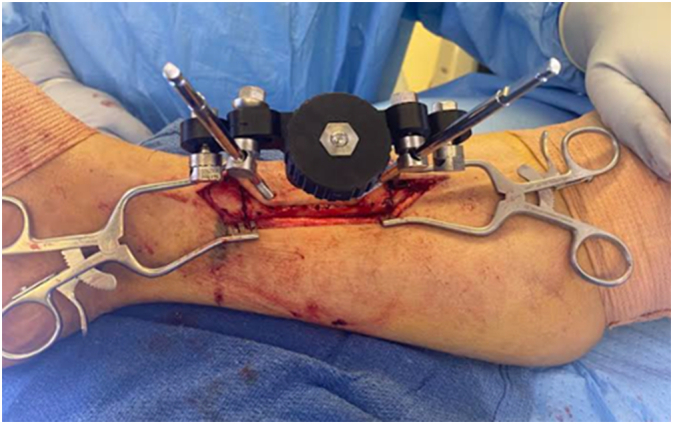
Placement of the unilateral external fixation device.

**Fig. 8:**
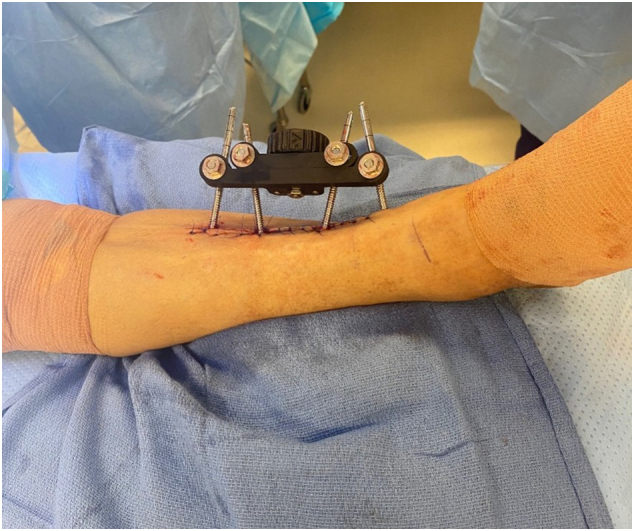
Stabilization of the fixator with two diverging 5.0 mm diameter bicortical half pins.

**Fig. 9:**
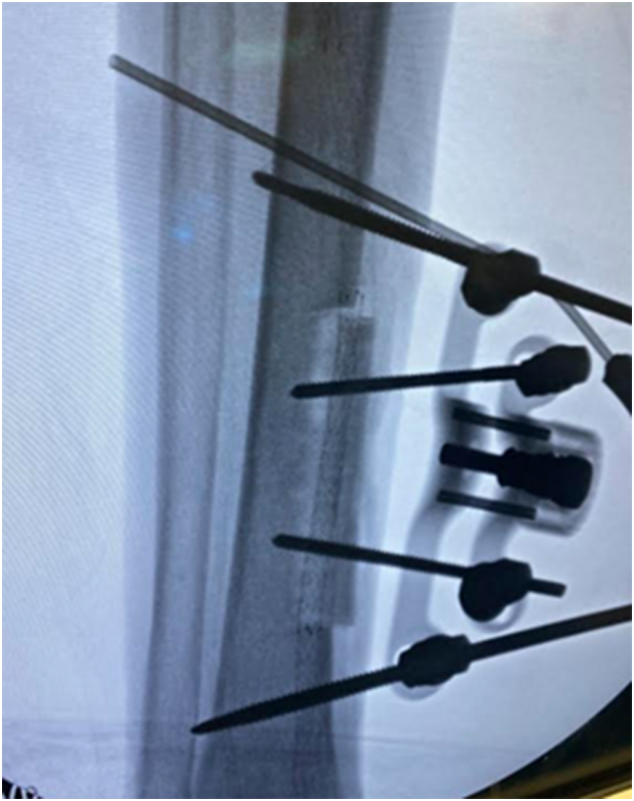
Fluoroscopic confirmation to verify osteotomy completion and correct functioning of bone transport device.

The unilateral external fixator was then attached to the bone transport half pins. This was followed by the placement of two 5.0 mm diameter diverging stabilization bicortical tibial half pins. Finally, the posterior aspect of the osteotomy was completed, and skin closure was performed (Fig. 7, Fig. 8, Fig. 9).

## 4 Postoperative protocol

**Fig. 10:**
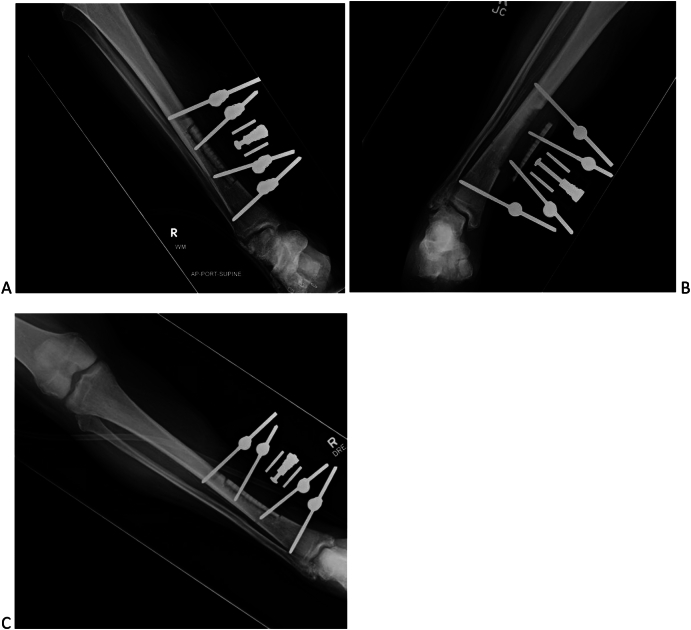
Radiographic dynamics of transverse tibial bone transport: A, latency period: B, distraction period; C, redocked transport segment.

A personalized post-operative plan was developed for the patient, addressing individual wound care and treatment needs. A 7-day latency period was given, followed by a daily distraction of 0.25 mm x2 per day for a total of 28 days, then 1.0 mm daily compression in four 0.25 mm increments for 14 days to reset the corticotomy (Fig. 10) [12]. After redocking, the external fixator was kept in place for an additional 8 weeks to achieve adequate bone healing.

During the distraction and compression periods, the patient was instructed to remain non-weight bearing, with progressive weight-bearing allowed until the final removal of the fixator. The patient was seen weekly, with radiographs obtained to monitor transport progression. Local wound care was performed weekly in the clinic as needed for pin site management.

During the postoperative course, the patient developed deep necrosis at the amputation site with exposed bone, likely due to residual ischemia. This necessitated a Lisfranc amputation, followed by allograft application, multiple surgical debridements, and wound vacuum therapy. The foot subsequently healed over a period of 6.5 months.

**Fig. 11:**
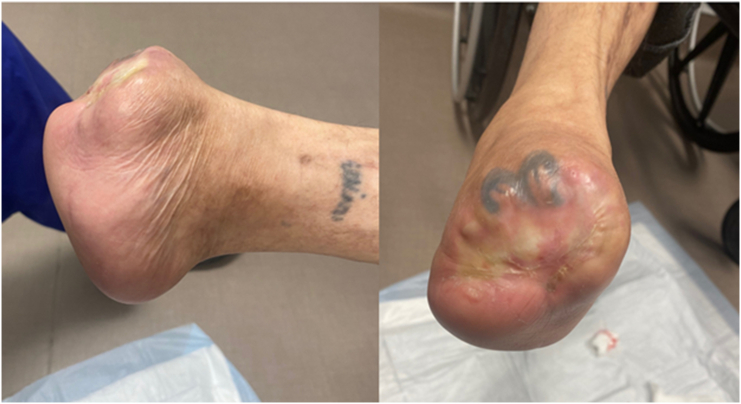
Fifteen months postoperative clinical photographs.

**Fig. 12:**
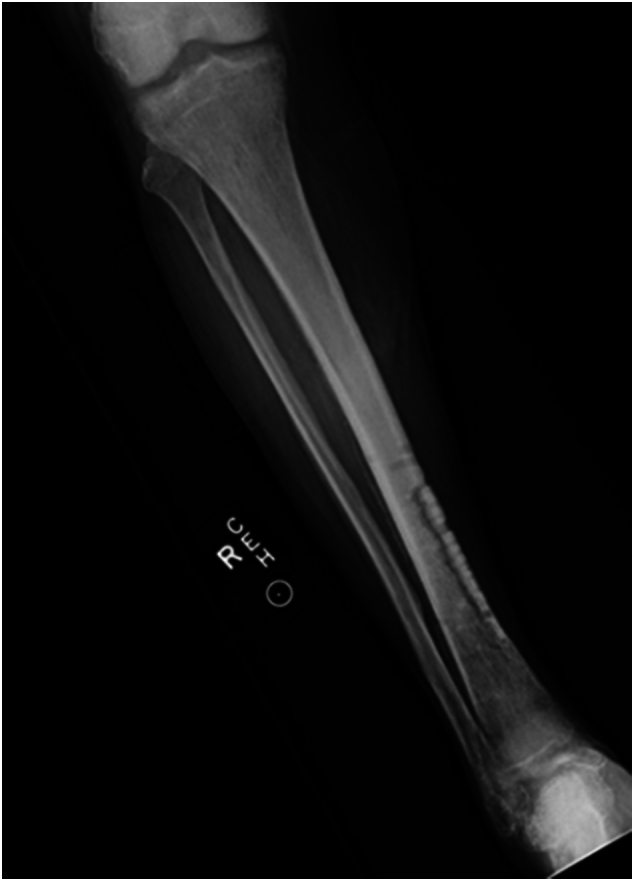
Final radiographs demonstrating healed bone transport segment.

**Fig. 13:**
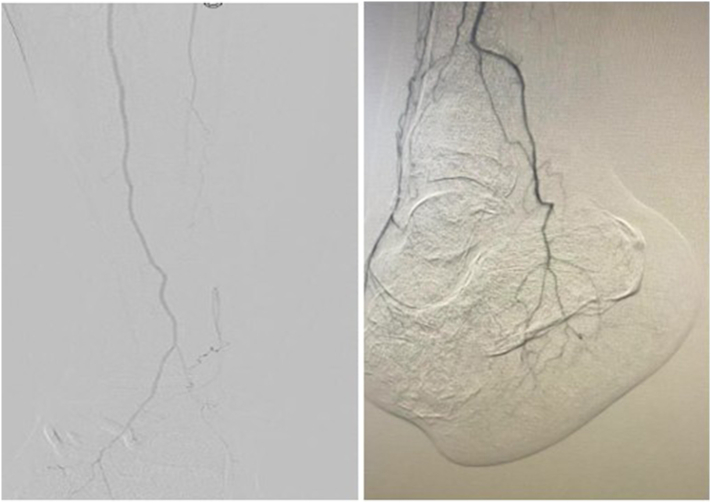
Comparative angiograms pre- and post-transverse tibial bone transport demonstrating improved distal tibial perfursion.

Fourteen months after the bone transport, a repeat angiogram performed by interventional radiology demonstrated improved distal perfusion with increased vascular reconstitution (Fig. 13). At her most recent clinical visit, 15 months post-procedure, the Lisfranc amputation stump was well-healed, and final radiographs showed excellent bone consolidation at the bone transport site (Fig. 11, Fig. 12). The patient is currently using an ankle-foot orthosis (AFO) for stability and assistance with ambulation.

In this case report, a transverse tibial bone transport technique in line with the SCARE criteria is described as a potential alternative for managing dysvascular feet with the goal of avoiding major below-the-knee amputations [13].

## 5 Discussion

Longitudinal tibial bone transport has emerged as a valuable surgical technique for the management of complex bone defects, particularly in the context of trauma, infection, and non-union. This method is grounded in the principles of distraction osteogenesis, wherein a bone segment is gradually distracted, allowing for the formation of new bone tissue to bridge the defect [[14], [15], [16]]. This technique not only addresses the loss of bone continuity but also enhances revascularization in the affected area, thereby promoting the overall healing process [6,7]. Based on experimental studies of transverse distraction by Ilizarov, the well-established tension-stress effect observed in longitudinal distraction was applied and evaluated in relation to neo-osteogenesis in the tibia. By day 14 of lateral distraction at a rate of 1 mm/day, the distraction gap became filled with a cellular network of osteoid-osseous trabeculae. Angiographic analysis confirmed the concurrent formation of new blood vessels within the distraction gap. Microscopic examination further revealed progressive capillary growth under the continued influence of tension-stress, resulting in a dense vascular network not only within the regenerating bone but also in the surrounding soft tissue. By day 20, this rapid angiogenesis led to a significantly greater number of blood vessels per unit area in the dermal tissue compared to the contralateral limb. These findings underscored the potential for clinical applications in treating circulatory disorders of the limbs [17]. Building upon these pioneering observations, Ilizarov demonstrated that distraction osteogenesis could be successfully applied not only in bone defect repair but also in the treatment of dysvascular limbs—a concept that continues to be validated by current research on the vascular benefits of transverse bone transport in ischemic pathologies [16,18,19].

A study conducted by Liu et al. examined the use of transverse tibial bone transport in patients with diabetic foot ulcers and peripheral vascular disease [20]. The researchers concluded that distraction osteogenesis combined with appropriate vascular interventions could reduce amputations effectively and overall improving outcomes for patients with chronic ischemic conditions. This concept of transverse tibial bone transport was further supported by Zhang et al. in 2021 who reported a significantly higher rate of limb salvage for those who were treated with this neovascularization promoting option with bone transport and demonstrated a reasonable alternative to amputation [21]. While Wang et al. reported a high success rate with bone transport, they found similar results to our case, noting that the wounds often required additional interventions for surgical wound care. They emphasized the importance of careful patient selection and the need for adjunctive therapies, such as revascularization or local flap coverage, to enhance healing outcomes [22]. The study by Chen et al. further examined the efficacy of transverse tibial bone transport in patients with critical limb ischemia complicated by diabetic foot ulcers [23]. This study was particularly focused on evaluating the combined effect of bone transport and vascular interventions on limb salvage rates. The findings suggested that transverse tibial bone transport, when combined with surgical revascularization, led to a significant reduction in amputation rates and improved healing outcomes. Moreover, Chen's study emphasized the need for a multidisciplinary approach, highlighting that successful limb salvage required not only surgical intervention but also careful management of underlying conditions like diabetes and peripheral arterial disease (PAD. This study underscores the importance of integrated care in optimizing outcomes for patients undergoing bone transport procedures. Despite its benefits, transverse tibial bone transport is not without challenges. The technique requires meticulous surgical planning and execution, as well as careful post-operative management. Complications such as nonunion, infection, and malalignment related to the corticotomy need to be considered. Most studies have shown that serial wound debridements and the potential need for allograft application are common and should be expected [[21], [22], [23], [24], [25]]. Our findings are consistent with these reports, with a healing time of nearly 7 months. This is comparable to the study by Yuan et al. in China, which followed 201 cases of ischemic diabetic foot ulcers reporting average 4.6 ± 1.6 months. While international case reports have demonstrated the effectiveness of transverse tibial bone transport in limb salvage, its application in the United States remains limited [[16], [17], [18], [19], [20], [21], [22], [23], [24], [25]]. Moreover, the literature lacks evidence comparing distal versus proximal transverse tibial bone transport, although proximal approaches have been extensively studied. In studies on distal tibial longitudinal distraction osteogenesis, Siddiqui reported a 93.3 % union rate in patients with Charcot neuroarthropathy when distal distraction was combined with ankle fusion, attributing the enhanced arthrodesis healing to increased blood flow at the corticotomy site [26]. Similarly, Chappell et al. observed an 83 % union rate in a mixed pathology cohort undergoing distal tibial distraction. While these outcomes are comparable to those seen with proximal distraction, no studies to date have directly compared distal transverse tibial distraction osteogenesis to its proximal counterpart [27]. In our case, we propose that utilizing a distal tibial corticotomy, rather than a proximal one, may create a prolonged hypervascular environment in the distal lower extremity, thereby enhancing neovascularization. This hypothesis is supported by our findings, with pre- and post-procedure angiograms (Fig. 13) demonstrating a markedly improved vascular network supplying the foot following distal transverse tibial bone transport. Our results suggest that this approach may contribute meaningfully to limb salvage efforts in dysvascular limbs and help reduce the incidence of below-knee amputations.

Further research is needed to optimize surgical technique, refine patient selection criteria, and better understand the timing and duration of transverse bone transport. Importantly, comparative studies are warranted to evaluate outcomes between proximal and distal corticotomy sites. As our understanding of tissue regeneration and neovascularization continues to evolve, the role of transverse tibial bone transport may expand, offering improved outcomes for patients with complex lower extremity pathologies—including non-unions, failed fusions, and chronic diabetic ulcerations.

Additionally, future investigations into the use of advanced vascular imaging modalities, both pre- and post-operatively, may provide valuable insights into perfusion changes and long-term vascular remodeling.

By building upon foundational studies and addressing current limitations, transverse tibial bone transport has the potential to become a cornerstone of limb salvage strategies—improving quality of life and reducing the need for major amputations in patients with complex limb-threatening conditions.

## 6 Conclusion

In conclusion, transverse tibial bone transport presents a promising approach for limb salvage in patients with ischemic feet, particularly in lower extremity reconstruction. This technique has demonstrated effectiveness in reducing the need for major amputations and improving clinical outcomes in patients at considerable risk of limb loss. The present case report involved a patient with poor healing potential below the ankle and forefoot gangrene, who achieved healing of a distal amputation stump with the aid of transverse tibial bone transport. These results are encouraging for the management of high-risk patients with diabetic foot and peripheral vascular disease (PVD). Continued advancements in understanding tissue regeneration and neovascularization, along with ongoing research into the efficacy of transverse tibial bone transport, will be crucial for refining this method and expanding its applicability to a broader range of patient populations.

## Author contribution

Dr. Estes – data collection, data analysis, writing the paper, literature review.

Dr. Samchukov – study concept/design, writing the paper, data interpretation.

## Consent

Written informed consent was obtained from the patient for publication of this case report and accompanying images. A copy of the written consent is available for review by the Editor-in-Chief of this journal on request.

## Ethical approval

Ethical Approval exemption from Western University of Health Sciences. Reason: Case reports or case series of three or less individuals are not considered Human Subject's Research therefore IRB review is not required.

This study did not require ethics committee approval as it involved only publicly available data with no identifiable personal information, therefore posing minimal risk to participants.

## Guarantor

Dr. Estes.

## Research registration number

1. Name of the registry: researchregistry.knack.com
2. Unique identifying number or registration ID: researchregistry11073
3. Hyperlink to your specific registration (must be publicly accessible and will be checked): https://researchregistry.knack.com/researchregistry#home/registrationdetails/67be12612b1db00305619b34/.

## Declaration of Generative AI and AI-assisted technologies in the writing process

During the preparation of this work, the author used Grammarly and Jenni.AI to format writing, and create consistent, standardized output and organize the research content. After using these tools, the corresponding author reviewed and edited the content.

## Funding

Orthofix.

## Declaration of competing interest

Consultant, R&D – Dr. Samchukov Orthofix Sea Spine.

Consultant – Dr. Estes Orthofix Sea Spine.
